# Proton gradient controls the lateral rearrangement of inner membrane domains in response to membrane fluidizer stress in *Mycobacterium smegmatis*

**DOI:** 10.1016/j.jbc.2025.110361

**Published:** 2025-06-11

**Authors:** Malavika Prithviraj, Shivangi, Joel S. Freundlich, Yasu S. Morita

**Affiliations:** 1 Department of Microbiology, University of Massachusetts, Amherst, Massachusetts, USA; 2 Department of Pharmacology, Physiology, and Neuroscience, Rutgers University – New Jersey Medical School, Newark, New Jersey, USA

**Keywords:** membrane fluidity, *Mycobacterium*, plasma membrane, proton motive force, stress response

## Abstract

*Mycobacterium smegmatis* partitions its plasma membrane into two distinct regions: the inner membrane domain (IMD) and the conventional plasma membrane. IMD, enriched in the sub-polar regions of actively growing rod-shaped cells, contains many membrane proteins involved in cell envelope biosynthesis. Dibucaine, a membrane fluidizer, disrupts plasma membrane integrity and de-partitions the IMD from the subpolar regions. We do not know what governs the de-partitioning of the IMD in response to dibucaine stress. In this study, we investigated the stress response of the IMD under respiration defect. We first depleted MenG, a key enzyme in the menaquinone biosynthesis, by CRISPRi and observed that the IMD does not respond to dibucaine-induced membrane stress. CRISPRi-induced knockdown of *qcrC*, a gene encoding a component of an electron transport chain cytochrome, corroborated the results of *menG* knockdown. In contrast, neither CRISPRi knockdown of *atpD*, a gene encoding a component of the ATP synthase nor inhibition of ATP synthase by bedaquiline inhibited the dibucaine-induced de-partitioning of sub-polar IMD as robustly as CRISPRi knockdowns of *menG* and *qcrC*. Pretreatment with the protonophore carbonyl cyanide *m*-chlorophenyl hydrazone (CCCP) prevented dibucaine-induced IMD de-partitioning. Furthermore, pretreatment with nigericin, which acts as an H^+^/K^+^ antiporter and disrupts the proton gradient without affecting membrane potential, also inhibited the IMD de-partitioning in a way similar to CCCP. Taken together, our findings suggest that membrane stress-induced IMD delocalization is not a passive lipid dispersion but an active membrane rearrangement dependent on an electrochemical gradient of the proton.

Bacteria compartmentalize their plasma membrane to meet the demands of many functions it must play. Mycobacteria grow from polar ends and create a sub-polar membrane domain termed the inner membrane domain (IMD) (1), presumably to support the polarly enriched cell envelope elongation (2). Biochemically, the IMD is purified as a pure plasma membrane without cell wall components. This is in contrast to the conventional plasma membrane, which is co-purified with the tightly associated cell wall and is termed PM-CW (3, 4). The IMD carries numerous enzymes essential for cell envelope biosynthesis and localizes to the subpolar regions of the cell during active growth (3, 4, 5, 6, 7, 8). Under stress conditions, such as exposure to sublethal concentrations of membrane fluidizers and antibiotics, IMD-associated proteins delocalize from the subpolar region and form patches along the side wall of the cell (1, 5, 9).

Genome-wide transposon sequencing revealed genes essential for surviving and recovering from dibucaine-induced membrane fluidization stress (1, 10), and the gene *cfa*, which encodes cyclopropane fatty acyl synthase, was identified to be critical in this process. Cfa is involved in the synthesis of tuberculostearic acid, a branched-chain fatty acid (C19:0), from unsaturated oleic acid (C18:1). A deletion mutant, Δ*cfa*, is devoid of tuberculostearic acid-containing phospholipids and instead accumulates oleic acid-containing phospholipids. Unsaturated fatty acids such as oleic acid fluidize the membrane, and excess enrichment of oleic acid will likely make the plasma membrane of Δ*cfa* defective in homeoviscous adaptation. Proposed by Sinensky in 1974, homeoviscous adaptation is a widely observed phenomenon in biology, in which cell membrane lipid composition changes in response to environmental cues to maintain adequate membrane fluidity (11). Defective homeoviscous adaptation due to the lack of tuberculostearic acid biosynthesis appears to make the sub-polar re-partitioning of the IMD after dibucaine treatment less efficient. While our study on Cfa provided insights into the genes necessary for plasma membrane re-partitioning and implied that the sub-polar re-partitioning of the IMD after dibucaine treatment requires optimal membrane fluidity, it remains elusive what governs the initial de-partitioning of the IMD during dibucaine treatment.

In this study, we unexpectedly found that the de-partitioning of the IMD upon dibucaine treatment requires a proton gradient across the plasma membrane. We show that an electrochemical gradient of the proton is required for the dibucaine-induced IMD de-partitioning.

## Results

### Transcriptional repression of respiratory chain enzymes prevents the IMD from de-partitioning under dibucaine stress

**Figure 1:**
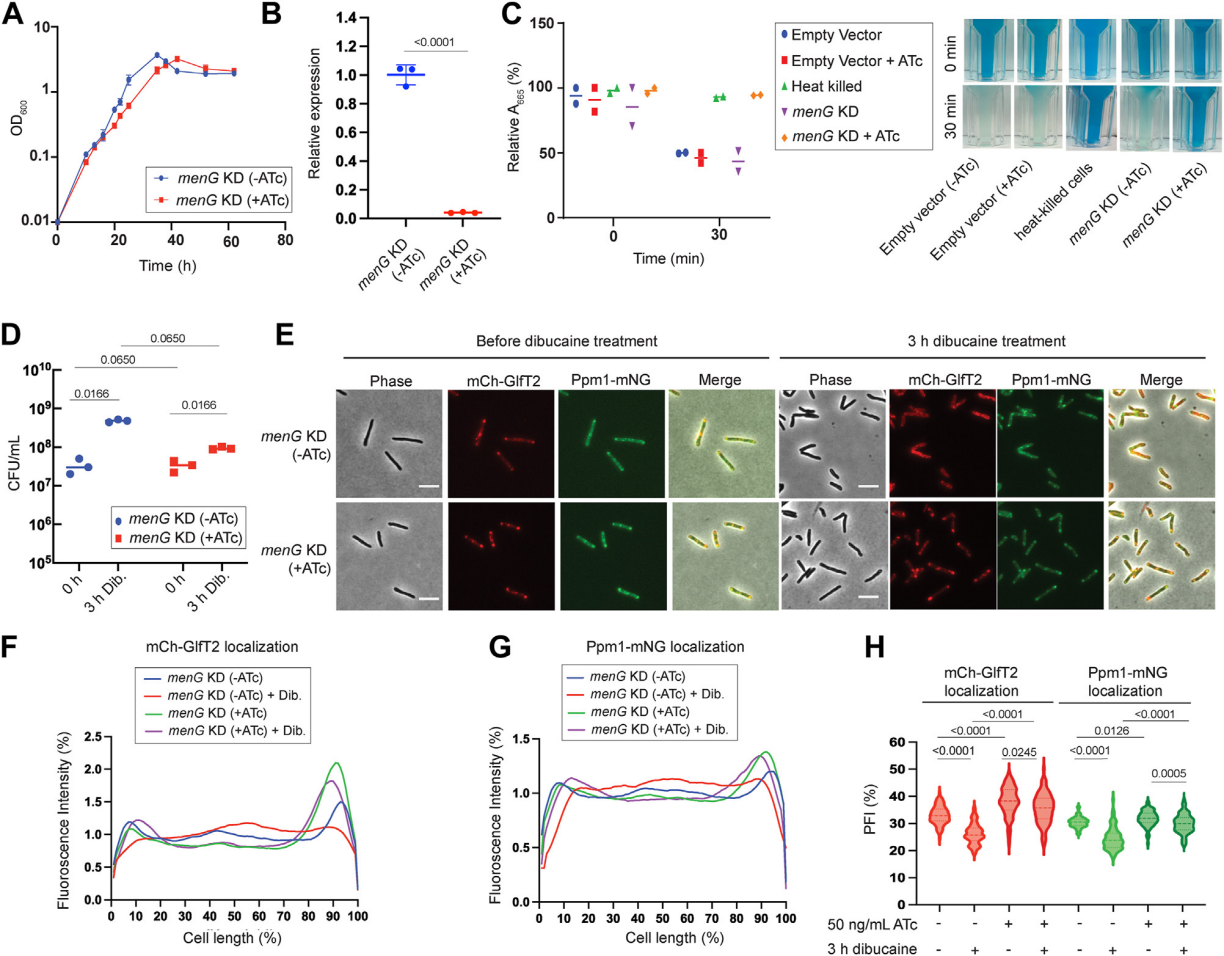
**Transcriptional repression of menaquinone biosynthesis prevents the IMD from de-partitioning under dibucaine stress**. *A*, growth curve of *menG* knockdown (KD) strain in the presence and absence of 50 ng/ml anhydrotetracycline (ATc), showing that *menG* depletion (+ATc) causes growth delay. *B*, *menG* transcript level after 20 h incubation with and without 50 ng/ml treatment of ATc normalized to reference gene *gyrB. p* values were determined by unpaired *t* test. *C*, oxygen consumption by *menG* KD strain with and without 20-h treatment with 50 ng/ml of ATc. Decolorization of *methylene blue* indicates oxygen consumption. *Methylene blue* was added to the cell culture and the A_665_ of *methylene blue* was monitored for 30 min. Oxygen consumption was expressed as a ratio of A_665_ at 0 min to A_665_ at 30 min. Heat killed cells and cells carrying an empty CRISPRi vector were used as negative and positive controls. *D*, colony-forming units (CFUs) were calculated to determine the survival of *menG* KD strain during 3-h treatment with 200 μg/ml dibucaine. Cells are grown for 20 h in the presence of ATc prior to the addition of dibucaine. We have consistently observed an increase in CFU after dibucaine treatment. We have observed similar effects of dibucaine and benzyl alcohol on CFU previously (10, 43), and speculate that these membrane fluidizers may disperse clumpy cells, leading to increased CFU. *p* values were determined using two-way analysis of variance (ANOVA) followed by Tukey's multiple comparison test. *E*, effects of dibucaine treatment on IMD marker proteins with or without *menG* depletion. IMD markers, mCherry-GlfT2 and Ppm1-mNeonGreen, remain polarly localized under dibucaine stress when *menG* is depleted (+ATc). Scale bars, 5 μm. *F* and *G*, fluorescence intensity profiles of mCherry-GlfT2 (*F*) and Ppm1-mNeonGreen (*G*) quantified by Oufti. N = 100 for all conditions. *H*, *menG* depletion made the IMD stuck at the subpolar region under dibucaine stress. The percentage of signal associated with the distal 15% of rod-shaped cells was quantified as polar fluorescence index (PFI). *p* values were determined by Kruskal-Wallis test followed by Dunn's multiple-comparison test.

To assess the impact of respiratory defects on the IMD dynamics in mycobacteria, we first repressed the transcription of several essential respiratory genes by CRISPR interference (CRISPRi) (12). First, we tested *menG*, which encodes demethylmenaquinone methyl transferase, an enzyme involved in the final maturation steps of menaquinone biosynthesis (13). We previously showed that *menG* cannot be deleted from the *Mycobacterium smegmatis* genome unless there is an extrachromosomal copy of *menG* available on a tet-off expression vector (13). When anhydrotetracycline (ATc) was added, the depletion of MenG led to the accumulation of its substrate, demethylmenaquinone, and the growth was progressively slowed down over the course of three sub-culturing, further supporting the essentiality of the gene (13). In support, *menG* is predicted to be essential in *M. smegmatis* by a genome-wide Tn-seq study (14) and by a CRISPRi screening, with a vulnerability index of −4.113, which falls in the top 7% of the most vulnerable genes in the genome-wide screening (12). We obtained the CRISPRi plasmid, pMSMEG_1115.sgRNA, from the MSRdb, and electroporated it into our established IMD marker strain, expressing fluorescent protein fusions of two IMD-associated proteins, mCherry-GlfT2 and Ppm1-mNeonGreen, from their respective chromosomal loci (3). When CRISPRi was induced by the addition of ATc, growth started to delay within 20 h (Fig. 1*A*). To examine the transcriptional knockdown of *menG*, we took the 20-h time point and determined the *menG* transcript levels using quantitative real-time PCR (qRT-PCR). In biological triplicate, *menG* transcription was suppressed 24 times upon CRISPRi induction relative to negative control (no ATc addition), confirming an effective transcriptional knockdown (Fig. 1*B*). We further tested the oxygen consumption activity by colorimetric methylene blue assay at the same 20-h time point. We found that the rate of oxygen consumption was significantly reduced when the cells were treated with ATc compared with no ATc control (Fig. 1*C*). Taken together, these prior and current studies indicate that MenG is a key enzyme for aerobic respiration in mycobacteria.

Next, we treated the *menG*^CRISPRi^ mCherry-GlfT2 Ppm1-mNeonGreen strain with ATc for 20 h to deplete *menG*, and then challenged with dibucaine for 3 h to fluidize the membrane. With or without ATc-induced *menG* depletion, dibucaine did not kill the cells as measured by colony-forming units (CFU) (Fig. 1*D*). In the absence of *menG* depletion, both IMD markers were sub-polarly enriched prior to the dibucaine challenge. After dibucaine treatment, as previously observed (1, 10), both IMD markers were redistributed throughout the cell and sub-polar IMD enrichment was disrupted (Fig. 1*E*). Fluorescence intensity profiles along the cell length quantitatively indicated the dissipation of the sub-polar fluorescence peaks of both mCherry-GlfT2 and Ppm1-mNeonGreen upon dibucaine challenge (Fig. 1, *F* and *G*). We also defined the sub-polar fluorescence index (PFI) of 100 cells as described earlier (1) (Fig. 1*H*). The PFI distribution confirmed that the sub-polar fluorescence enrichment of IMD proteins was diminished upon the dibucaine challenge. In striking contrast, when *menG* was depleted, both IMD markers remained enriched at the pole even after dibucaine treatment (Fig. 1, *E*–*H*), suggesting that a respiratory defect prevents sub-polarly enriched IMD proteins from being redistributed.

**Figure 2:**
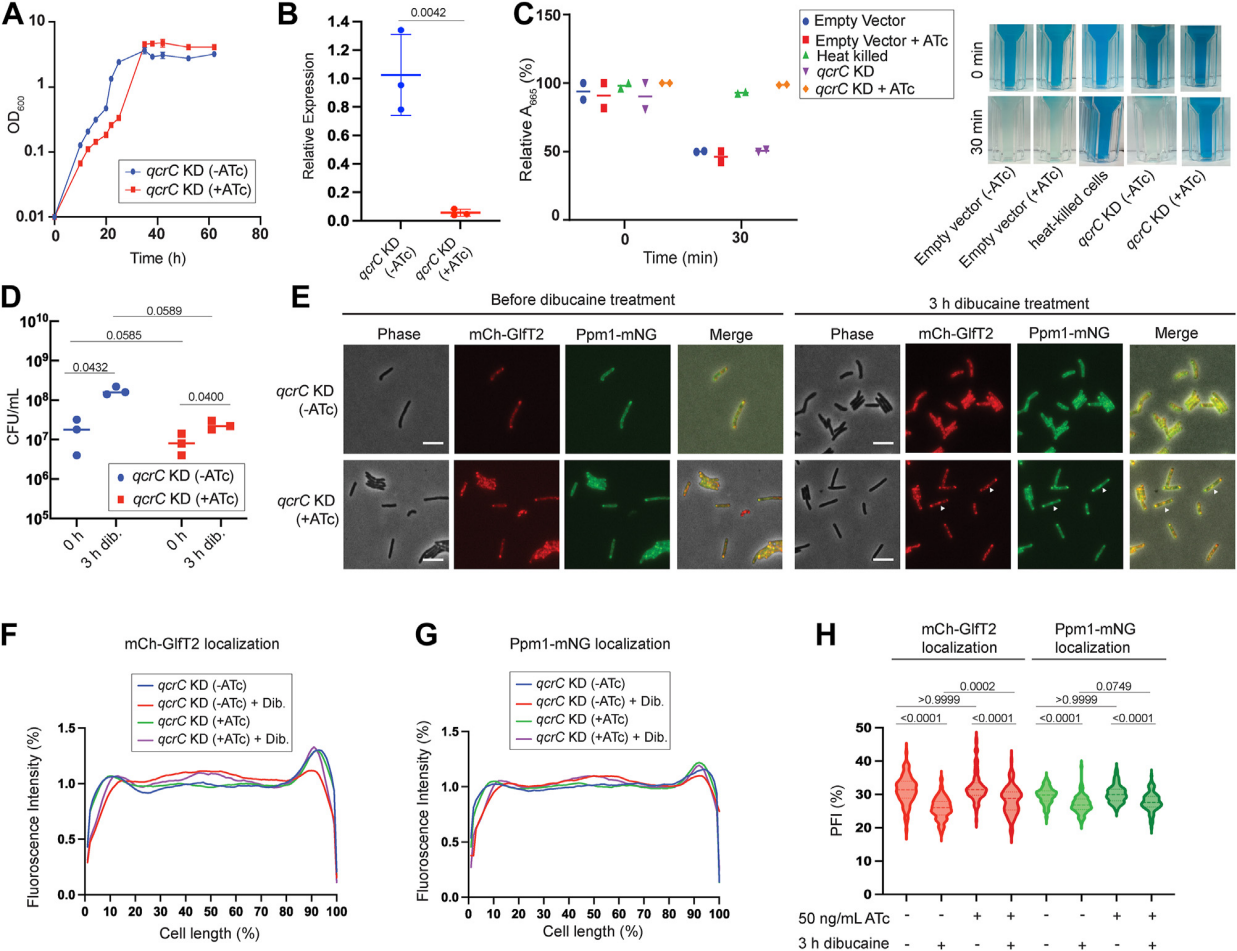
**CRISPRi knockdown of *qcrC* prevents the IMD from de-partitioning under dibucaine stress**. *A*, growth curve of *qcrC* knockdown (KD) strain with or without 50 ng/ml ATc. Growth delays when *qcrC* is depleted. *B*, *qcrC* transcript level after 20 h incubation with and without 50 ng/ml treatment of ATc normalized to reference gene *gyrB. p* values were determined by unpaired *t* test. *C*, Oxygen consumption by *qcrC* KD strain with and without 16-h ATc treatment. See Figure 1 legend for details. Data from heat-killed cells and cells carrying an empty CRISPRi vector control are reproduced from Figure 1*C* as this experiment was done together with Figure 1*C* experiment. *D*, CFUs were calculated to determine the survival of *qcrC* KD strain during 3-h dibucaine treatment. *p* values were determined using two-way ANOVA followed by Tukey's multiple comparison test. *E*, effects of dibucaine treatment on IMD markers, mCherry-GlfT2 and Ppm1-mNeonGreen, with or without *qcrC* knockdown. IMD de-partitioning under dibucaine stress was blocked when *qcrC* was knocked down. *Arrowhead*, sidewall punctate. Scale bars, 5 μm. *F* and *G*, fluorescence intensity profiles of mCherry-GlfT2 (F) and Ppm1-mNeonGreen (G) were quantified by Oufti. N = 100. *H*, *qcrC* depletion led to less pronounced redistribution of IMD even after dibucaine treatment. See Figure 1 legend and methods for PFI calculation. *p* values were determined by Kruskal-Wallis test followed by Dunn's multiple-comparison test.

To confirm the effect of the respiratory defect on IMD dynamics, we next suppressed the transcription of *qcrC* by CRISPRi using pMSMEG_4261.sgRNA. QcrC is a subunit of the cytochrome bc1 complex and *qcrC* is the first gene of the *qcrCAB* operon (15). The deletion of the *qcrCAB* operon causes severe growth defects in *M. smegmatis* (16) and the growth defect phenotype was corroborated by a genome-wide Tn-seq study (14). With a vulnerability index of −3.530 (top 8% most vulnerable), *qcrC* was also labeled as CRISPRi-essential (12). Indeed, CRISPRi knockdown of *qcrC* in the mCherry-GlfT2 Ppm1-mNeonGreen dual IMD marker strain confirmed substantial growth delay (Fig. 2*A*). At the 20-h time point, the *qcrC* transcript was reduced 18 times in ATc-treated cells compared with untreated cells, confirming effective CRISPRi knockdown (Fig. 2*B*). Oxygen consumption was also reduced (Fig. 2*C*), being consistent with the critical role of the cytochrome bc1 in aerobic respiration. We confirmed that the *qcrC*^CRISPRi^ dual IMD marker cells remained viable after the dibucaine challenge even when *qcrC* was knocked down for 16 h (Fig. 2*D*). Similar to *menG* knockdown, dibucaine-induced de-partitioning of the two sub-polar IMD marker proteins was diminished when *qcrC* CRISPRi was induced by ATc (Fig. 2*E*). Fluorescence intensity profiles across the cell length indicated that dibucaine did not mobilize the sub-polarly enriched IMD in knockdown cells (Fig. 2, *F* and *G*). The PFI did not show the inhibitory effect of *qcrC* knockdown as prominently as the fluorescence length profile (Fig. 2*H*). This appeared to be due to the *qcrC* knockdown cells displaying some distribution of IMD proteins in the mid-cell regions as puncta (Fig. 2*E*, arrowhead). Taken together with the *menG* knockdown results, these data suggest that active oxidative phosphorylation is important for the dynamic rearrangement of the IMD in response to membrane stress caused by the membrane fluidizer dibucaine.

### Inhibition of ATP synthesis does not prevent dibucaine-induced IMD departitioning

**Figure 3:**
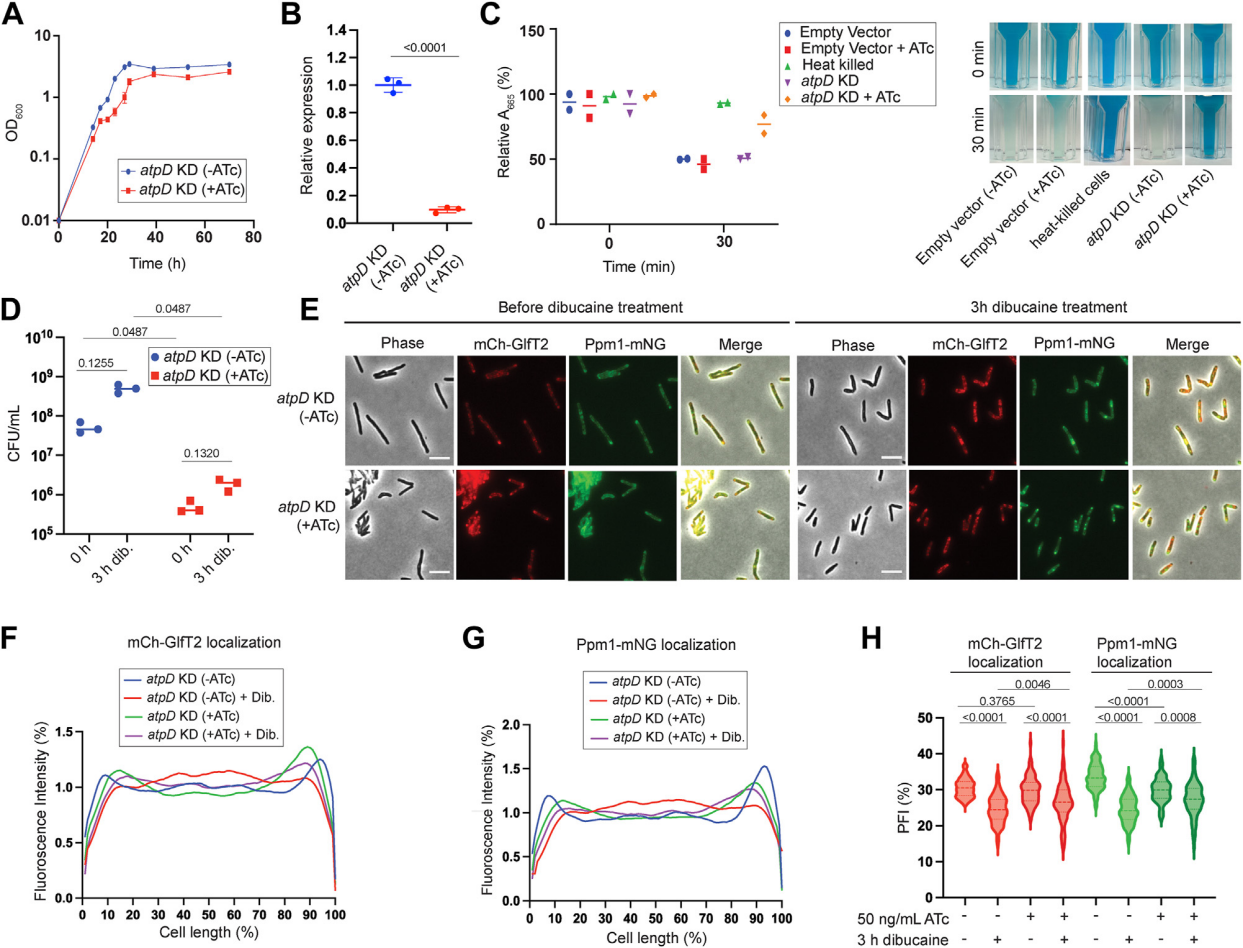
**IMD markers de-partition in response to dibucaine stress under CRISPRi knockdown of *atpD***. *A*, growth curve of *atpD* knockdown (KD) strain with and without 50 ng/ml ATc, showing a growth defect upon *atpD* depletion (+ATc). *B*, *atpD* transcript levels after 20 h incubation with and without 50 ng/ml ATc normalized to *gyrB. p* values were determined by unpaired *t* test. *C,* Oxygen consumption by *atpD* KD strain after 16 h of ATc treatment. See Figure 1 legend for details. Data from heat killed cells and cells carrying an empty CRISPRi vector control are reproduced from Figure 1*C* as this experiment was done together with Figure 1*C* experiment. *D*, CFUs were calculated to determine the survival of *atpD* KD strain during 3-h dibucaine treatment. *p* values were determined using two-way ANOVA followed by Tukey's multiple comparison test. *E*, effects of dibucaine treatment on IMD markers with and without *atpD* depletion. Scale bars, 5 μm. *F* and *G*, Fluorescence intensity profiles of mCherry-GlfT2 (*F*) and Ppm1-mNeonGreen (*G*) quantified by Oufti. N = 100. *H*, PFI with and without *atpD* depletion before and after dibucaine treatment. *p* values were determined by Kruskal-Wallis test followed by Dunn's multiple-comparison test.

A primary consequence of oxidative phosphorylation is the production of ATP. We wondered if ATP drives the IMD dynamics. We therefore knocked down *atpD*, the beta subunit of ATP synthase, in *M. smegmatis* to test the role of ATP synthesis. The genome-wide CRISPRi screening indicates that *atpD* is essential with the vulnerability index being −7.515 (12). We established the *atpD*^CRISPRi^ mCherry-GlfT2 Ppm1-mNeonGreen strain using pMSMEG_4936.sgRNA and demonstrated that CRISPRi induction leads to growth defects (Fig. 3*A*). Upon 20-h CRISPRi induction, *atpD* transcript levels were reduced by 10 times compared with untreated cells (Fig. 3*B*). At 16 h after CRISPRi induction, oxygen consumption was reduced (Fig. 3*C*). However, dibucaine did not affect the viability of cells after 3-h incubation (Fig. 3*D*), indicating that dibucaine is not bactericidal even when a key component of ATP synthase is depleted. Upon dibucaine treatment, two IMD markers de-partitioned from the sub-polar regions in the knockdown cells although de-partitioning was subtle, particularly for Ppm1-mNeonGreen (Fig. 3, *E*–*H*). Therefore, *atpD* knockdown was not as effective as *menG* and *qcrC* knockdowns in preventing dibucaine-induced IMD de-partitioning. These data suggested that IMD de-partitioning may be more dependent directly on the respiratory chain than ATP production.

**Figure 4:**
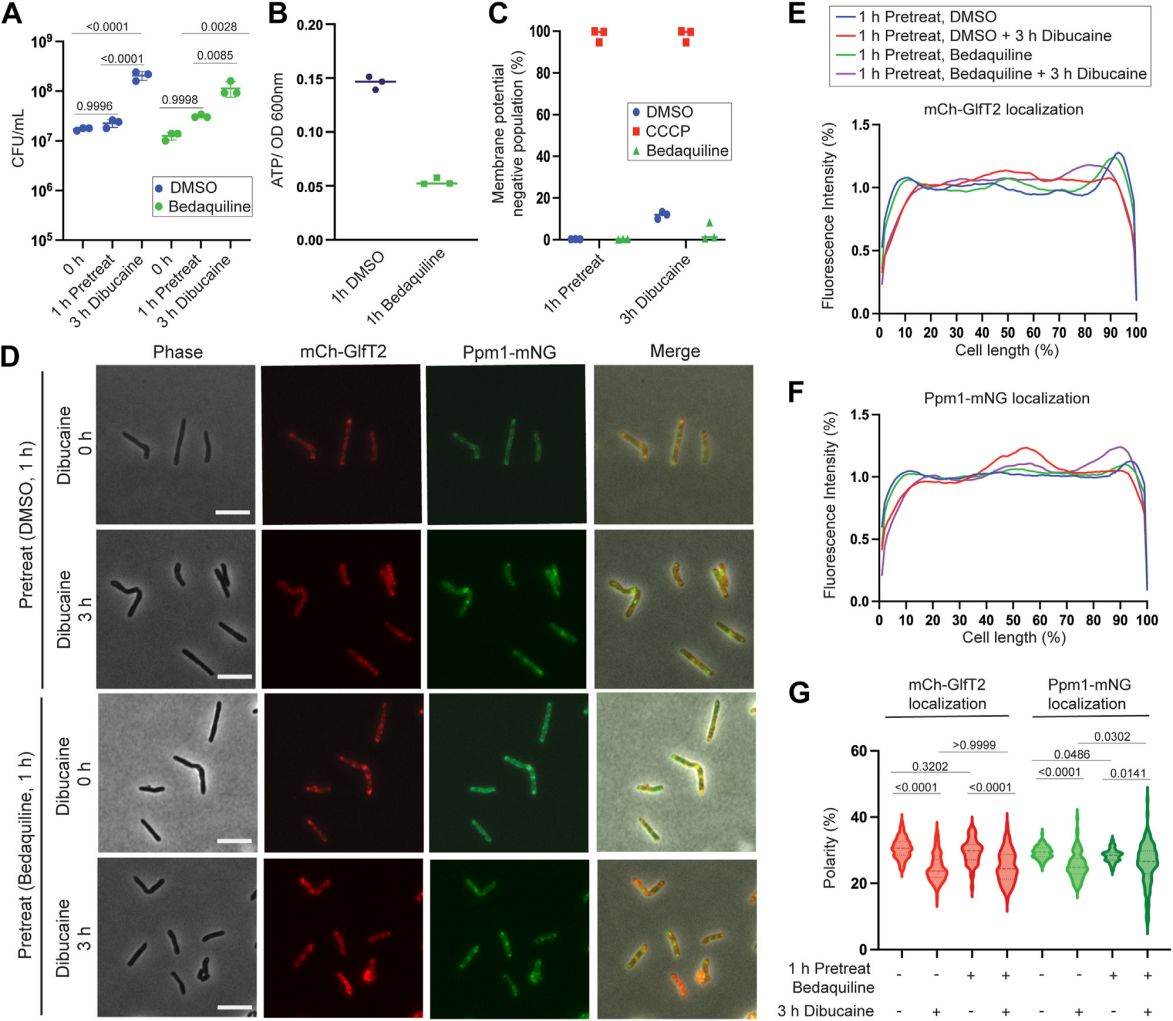
**Inhibition of ATP synthesis by bedaquiline does not prevent dibucaine-induced IMD de-partitioning**. *A*, CFU measurements of *M. smegmatis* after 1-h pretreatment with 14.4 nM bedaquiline followed by 3-h treatment with 200 μg/ml dibucaine, demonstrating the treatments are not bactericidal. *p* values were determined using two-way ANOVA followed by Tukey's multiple comparison test. *B*, cellular ATP content was measured by CellTiter-Glo 2.0 assay, a luciferase-based enzymatic assay. The ATP levels were reduced after a 1 h bedaquiline treatment. *C*, membrane potential analysis of cells treated with DiOC_2_(3) after 1-h treatment with bedaquiline followed by 3-h treatment with dibucaine. *Red/green* ratio of DiOC_2_(3) is the direct measurement of membrane potential and cells were analyzed by flow cytometry. Bedaquiline-treated cells maintained a positive membrane potential before and after dibucaine treatment. CCCP treatment served as a positive control for membrane potential disruption and DMSO treatment served as a negative control. *D*, effects of dibucaine treatment after bedaquiline pretreatment, demonstrating that IMD markers, mCherry-GlfT2 and Ppm1-mNeonGreen, delocalized from the poles upon dibucaine treatment. Scale bars, 5 μm. *E* and *F*, fluorescence intensity profiles of mCherry-GlfT2 (*E*) and Ppm1-mNeonGreen (*F*) cells quantified by Oufti. N = 100. *G*, PFI with and without bedaquiline treatment before and after dibucaine treatment. *p* values were determined by Kruskal-Wallis test followed by Dunn's multiple-comparison test.

Because the respiratory chain and ATP synthesis are tightly linked, prolonged 16-h incubation with ATc to induce *atpD* knockdown can have indirect impacts on cellular metabolism. To test the roles of ATP production more directly, we treated cells with bedaquiline, which is a tuberculosis drug initially identified through a screening of *M. smegmatis* (17) and inhibits mycobacterial ATPase (18). We treated the mCherry-GlfT2 Ppm1-mNeonGreen strain with 14.4 nM bedaquiline, a bacteriostatic concentration (19), for 1 h. We confirmed that this concentration of bedaquiline does not kill cells in 1-hour pretreatment (Fig. 4*A*) but reduced the cellular ATP concentration by ∼three times (Fig. 4*B*). Using 3,3′-diethyloxacarbocyanine iodide (DiOC_2_(3)) membrane potential probe, we confirmed that the membrane potential remained intact after bedaquiline treatment while carbonyl cyanide *m*-chlorophenyl hydrazone (CCCP), an ionophore that collapses the proton motive force (PMF), abrogated the membrane potential (Fig. 4*C*). Even after 3-h dibucaine treatment, the membrane potential remained intact in the presence of bedaquiline (Fig. 4*C*). We measured the intracellular pH using pH-sensitive ratiometric GFP and found that bedaquiline did not acidify the cytoplasm, indicating that the ΔpH was preserved during the 1-h pre-treatment and after 3 h of dibucaine treatment (Table 1). When we challenged the cells with dibucaine after 1-h bedaquiline treatment, the IMD markers were redistributed from the poles in a manner similar to dimethyl sulfoxide (DMSO) vehicle control pretreatment (Fig. 4, *D*–*F*). Polar index confirmed that sub-polar localization of both mCherry-GlfT2 and Ppm1-mNeonGreen was de-partitioned upon dibucaine treatment with or without bedaquiline pretreatment although the effect was marginal for Ppm1-mNeonGreen as it has been observed in *atpD* knockdown cells (Fig. 4*G*). Thus, both genetic and chemical inhibitions of ATP synthesis did not prevent the dynamic rearrangement of the IMD proteins in response to dibucaine-induced membrane stress as effectively as *menG* and *qcrC* knockdowns.

**Table 1:** Bedaquiline does not affect the intracellular pH

	Intracellular pH	
	DMSO	Bedaquiline
1 h Pre-Treatment	6.74 ± 0.05	6.88 ± 0.01
3 h Dibucaine Treatment	7.09 ± 0.01	7.13 ± 0.01

The cytoplasmic pH of cells before and after bedaquiline and dibucaine treatment was measured in a buffered medium (pH 6.65) using pH-sensitive ratiometric GFP. The internal pH of cells treated with 14.4 nM bedaquiline remains comparable to DMSO-treated cells, indicating an intact proton gradient.

### Dissipation of proton gradient prevents the redistribution of sub-polar IMD under dibucaine-induced membrane fluidization stress

**Figure 5:**
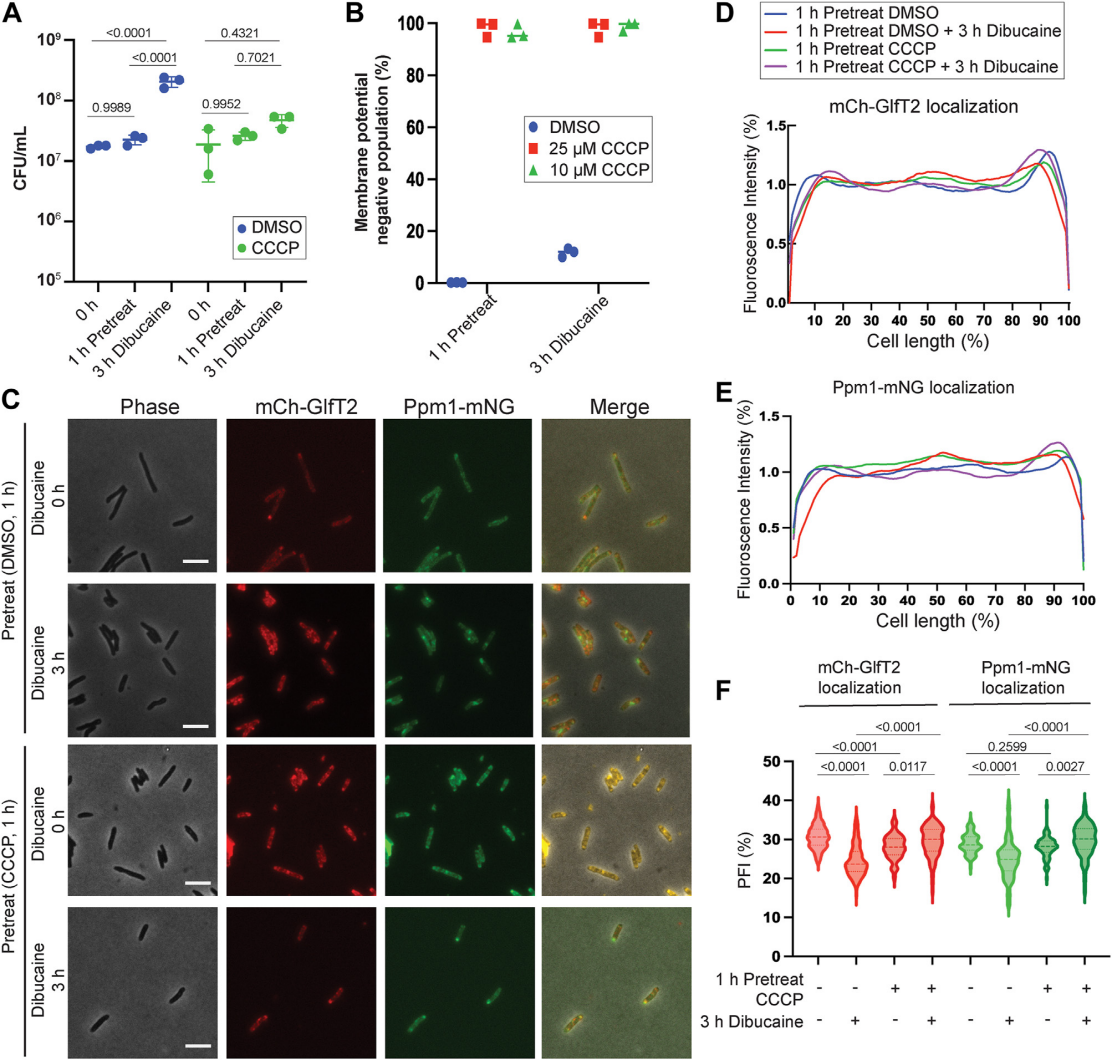
**Disruption of proton gradient prevents the de-partitioning of subpolar IMD under dibucaine-induced membrane stress**. *A*, CFUs, indicating the survival of *M. smegmatis* after 1-h treatment with 10 μM CCCP followed by 3-h treatment with 200 μg/ml dibucaine. Data from DMSO-treated vehicle control cells are reproduced from Figure 4*A* as this experiment was perfoemd together with Figure 4*A* experiment. *p* values were determined using two-way ANOVA followed by Tukey's multiple comparison test. *B*, flow cytometry analysis, indicating that 10 μM CCCP is sufficient to disrupt the membrane potential. Membrane potential was measured as described in Figure 4*C*. Data for CCCP and DMSO treatments were reproduced from Figure 4*C* as these two experiments were performed at the same time. *C*, effects of dibucaine treatment with or without CCCP pretreatment on two IMD markers, mCherry-GlfT2 and Ppm1-mNeonGreen. Both markers do not delocalize from the subpolar regions when cells were pretreated with CCCP. Scale bars, 5 μm. *D* and *E*, fluorescence intensity profiles of mCherry-GlfT2 (*D*) and Ppm1-mNeonGreen (*E*) cells quantified by Oufti. N = 100. *F*, PFI with and without CCCP treatment before and after dibucaine treatment. *p* values were determined by Kruskal-Wallis test followed by Dunn's multiple-comparison test.

Given the minimal effects of ATP synthesis inhibition on the IMD dynamics, we considered the possibility that *M. smegmatis* uses proton gradient directly to energize the IMD stress response. We treated the IMD dual marker strain with 10 μM CCCP. CCCP disrupts both proton gradient (ΔpH) and transmembrane potential (ΔΨ) across the plasma membrane. We confirmed that 10 μM CCCP is bacteriostatic and there is no reduction of cell viability after 3-h dibucaine treatment (Fig 5*A*). We used DiOC_2_(3), a fluorescent membrane potential probe, to demonstrate that CCCP disrupted membrane potential. Cells were treated with or without CCCP for 1 h and then with dibucaine for 3 h. Upon CCCP pretreatment, 98.0% of cells became membrane-potential-negative while 99.9% of cells were membrane-potential-positive without CCCP pretreatment (Fig. 5*B*). The proton gradient was measured using cells expressing a pH-sensitive ratiometric GFP as described. One hour pre-treatment with 10 μM CCCP acidified the intracellular pH to 6.40 when the pH of the external medium was 6.65, indicating a dissipation of the proton gradient (Table 2). Finally, we determined the impact of CCCP pretreatment on the dynamics of IMD in response to dibucaine. Both IMD marker proteins, mCherry-GlfT2 and Ppm1-mNeonGreen, remained in subpolar localization when the cells were pretreated with CCCP even after dibucaine treatment (Fig. 5, *C*–*F*). These results support the idea that the lateral movement of the IMD is driven by PMF.

**Table 2:** CCCP collapses ΔpH

	Intracellular pH	
	DMSO	CCCP
1 h Pre-Treatment	6.74 ± 0.05	6.40 ± 0.01
3 h Dibucaine Treatment	7.09 ± 0.01	6.72 ± 0.02

The cytoplasmic pH of cells before and after CCCP and dibucaine treatment was measured in a buffered medium (pH 6.65) using pH-sensitive ratiometric GFP. The pH of cells treated with 10 μM CCCP was reduced compared with DMSO-treated cells, indicating a collapsed proton gradient.

**Figure 6:**
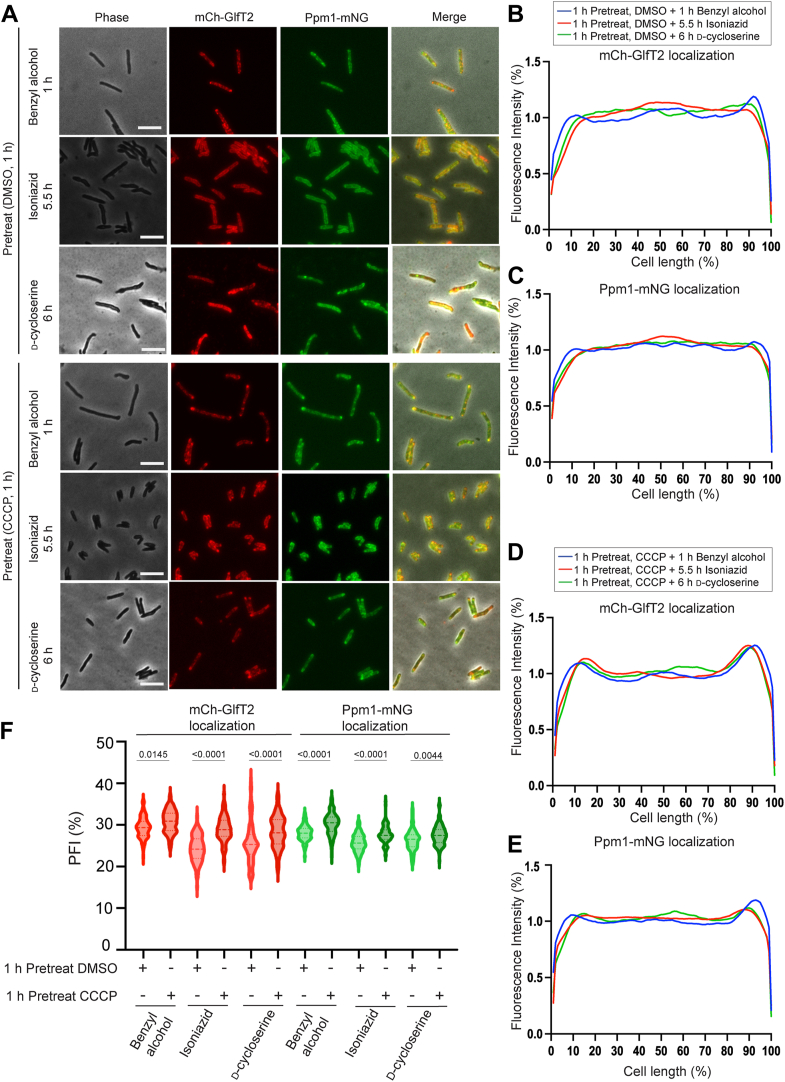
**Disruption of proton gradient prevents the de-partitioning of subpolar IMD under benzyl alcohol-induced membrane stress and other antibiotic stresses**. *A*, effects of 100 mM benzyl alcohol, 50 μg/ml isoniazid and 40 μg/ml d-cycloserine treatment with or without CCCP pretreatment on two IMD markers, mCherry-GlfT2 and Ppm1-mNeonGreen. Both markers do not delocalize from the subpolar regions when cells were pretreated with CCCP. Scale bars, 5 μm. *B–E*, fluorescence intensity profiles of mCherry-GlfT2 (*B* and *D*) and Ppm1-mNeonGreen (*C* and *E*) after stress exposure. Cells were pretreated with vehicle control (*B* and *C*) or CCCP (*D* and *E*). Fluorescence intensities were quantified by Oufti. N = 100. *F,* PFI with and without CCCP treatment before and after benzyl alcohol and antibiotics treatment. *p* values were determined by Kruskal-Wallis test followed by Dunn's multiple-comparison test.

IMD departitions from the subpolar region not only by dibucaine but also by other stressors including another membrane fluidizer benzyl alcohol (1, 5) as well as antibiotics such as d-cycloserine (9). We, therefore, tested if CCCP inhibits IMD redistribution in response to other stress conditions. In a vehicle control experiment, we pretreated cells with DMSO and challenged them with benzyl alcohol, isoniazid, or d-cycloserine (Fig. 6, *A*–*C*). As previously documented (1), mCherry-GlfT2 does not redistribute significantly after benzyl alcohol treatment for unknown reasons (Fig. 6, *B* and *F*). Apart from this sole exception, both mCherry-GlfT2 and Ppm1-mNeonGreen lost their subpolar enrichment and redistributed throughout the cell under all stress conditions tested (Fig. 6, *B*, *C*, and *F*). In contrast, 1 hour of CCCP pretreatment prior to these stress exposures diminished the delocalization of the IMD from the polar regions (Fig. 6, *D*–*F*). These results suggest that proton gradient-dependency of the stress-induced IMD departitioning is a general mechanism of stress response rather than a phenomenon specific to dibucaine.

### Dissipation of transmembrane potential does not affect dibucaine-induced membrane redistribution of the IMD

**Figure 7:**
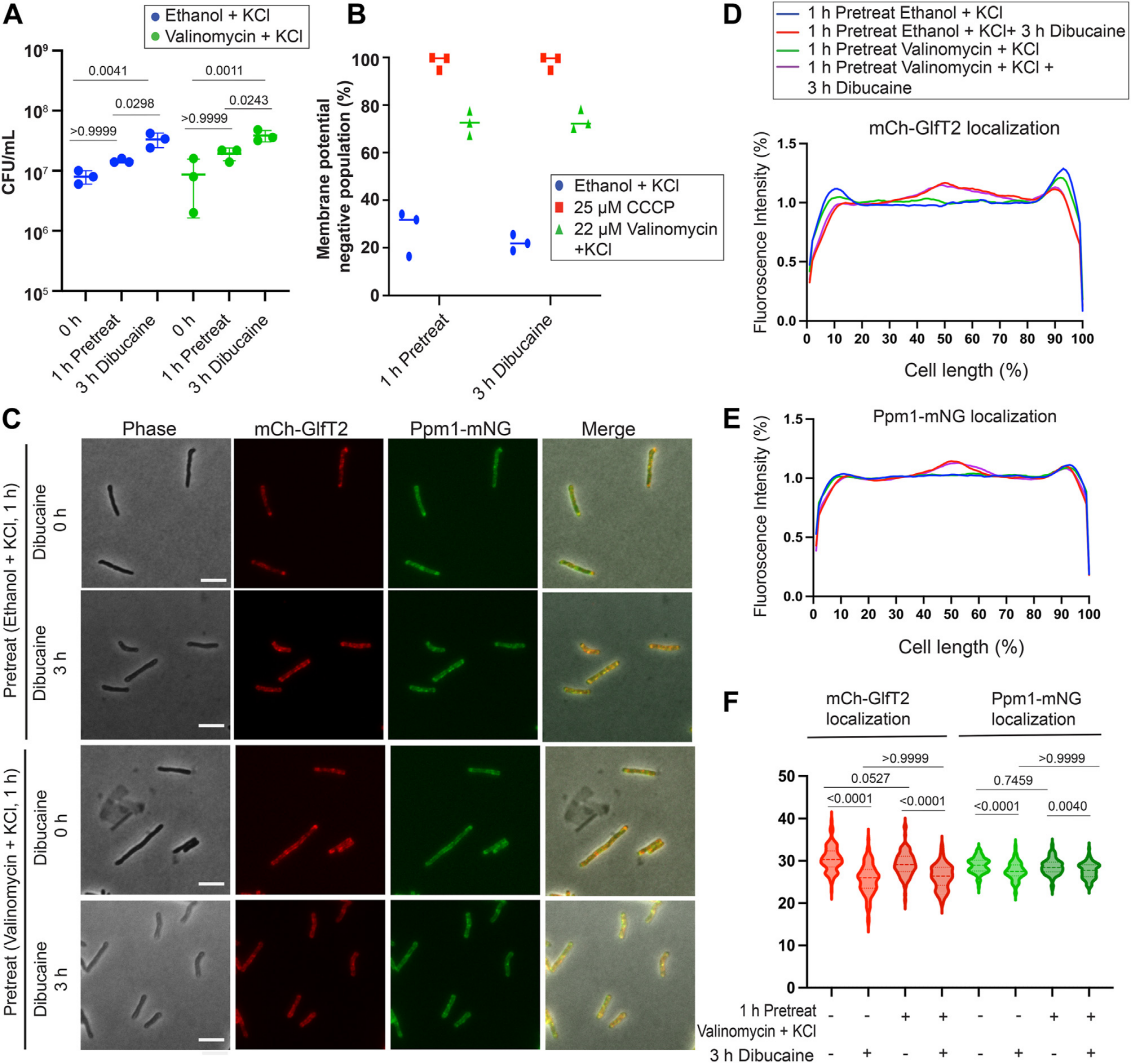
**Dissipation of transmembrane potential does not affect dibucaine-induced membrane redistribution of the IMD**. *A*, CFUs, indicating the survival of *M. smegmatis* after 1-h treatment with 22 μM valinomycin with 200 mM KCl followed by 3-h treatment with 200 μg/ml dibucaine. *p* values were determined using two-way ANOVA followed by Tukey's multiple comparison test. *B*, flow cytometry analysis, indicating that 22 μM valinomycin disrupts the membrane potential of about 75% of the cell population. Membrane potential was measured as in Figure 4*C*. *C*, effects of dibucaine treatment with or without valinomycin pretreatment on two IMD markers, mCherry-GlfT2 and Ppm1-mNeonGreen. mCherry-GlfT2 delocalized from the subpolar regions even after cells were pretreated with valinomycin. De-partitioning of Ppm1-mNeonGreen was subtle regardless of the valinomycin pretreatment (see text for details). Scale bars, 5 μm. *D* and *E*, fluorescence intensity profiles of mCherry-GlfT2 (*D*) and Ppm1-mNeonGreen (*E*) cells quantified by Oufti. N = 100. *F*, PFI with and without valinomycin treatment before and after dibucaine treatment. *p* values were determined by Kruskal-Wallis test followed by Dunn's multiple-comparison test.

As a protonophore, CCCP disrupts both aspects of the PMF, transmembrane potential (ΔΨ) and proton gradient (ΔpH). Therefore, we wanted to determine which aspect of the PMF is important for the de-partitioning of the IMD. To determine this, we utilized valinomycin, an ionophore that acts as a K^+^(potassium) ion-specific transporter. Valinomycin disrupts the K^+^ gradient across bacterial cell membranes, thereby dissipating the transmembrane potential without affecting ΔpH. We pretreated *M. smegmatis* cells with 22 μM valinomycin in the presence of 200 mM KCl, a previously established condition in mycobacteria (20), followed by 3 h of dibucaine treatment. Neither valinomycin pretreatment nor dibucaine treatment affected cell viability (Fig. 7*A*). Treatment with ethanol (vehicle control) in the presence of 200 mM KCl displayed a modest impact on membrane potential, where ∼30% of the cells lost their transmembrane potential. The addition of 22 μM valinomycin further depolarized the cells, causing ∼70% of the cell population to lose their membrane potential (Fig. 7*B*). To evaluate the impact of valinomycin on the proton gradient, a ratiometric pH-sensitive GFP strain was used to record the intracellular pH of the cells in an extracellular medium of pH 6.62. The proton gradient remained unaffected in both ethanol and valinomycin treated cells before and after dibucaine treatment (Table 3). We next examined the IMD localization of cells pretreated with valinomycin for 1 h. The polar localization of mCherry-GlfT2 was retained with or without valinomycin pretreatment. However, upon 3-h dibucaine treatment, the IMD was redistributed, with a majority of the signal shifting towards the side wall regions, particularly at the mid-cell (Fig. 7, *C* and *D*). For Ppm1-mNeonGreen, pretreatment with KCl alone made the IMD de-partitioning less pronounced for unknown reasons (Fig. 7, *C* and *E*). However, similar to mCherry-GlfT2, there is an enrichment of Ppm1-mNeonGreen at the mid-cell after dibucaine treatment (Fig. 7*E*). Importantly, we observed a similar dibucaine-induced enrichment of Ppm1-mNeonGreen at the mid-cell even when cells were pretreated with valinomycin. Together, these data support the notion that valinomycin is not as effective as CCCP in blocking subpolar IMD de-partitioning.

**Table 3:** Valinomycin collapses ΔΨ but retains ΔpH

	Intracellular pH	
	Ethanol + KCl	Valinomycin + KCl
1 h Pre-Treatment	6.86 ± 0.005	6.90 ± 0.02
3 h Dibucaine Treatment	7.08 ± 0.02	7.06 ± 0.03

The intracellular pH before and after valinomycin and dibucaine treatment was measured in a buffered medium (pH 6.62) using pH-sensitive ratiometric GFP. The internal pH of cells treated with 22 μM valinomycin with 200 mM KCl remain comparable to ethanol-treated cells, indicating an intact proton gradient.

### Proton gradient is critical for the dibucaine-induced IMD rearrangement

**Figure 8:**
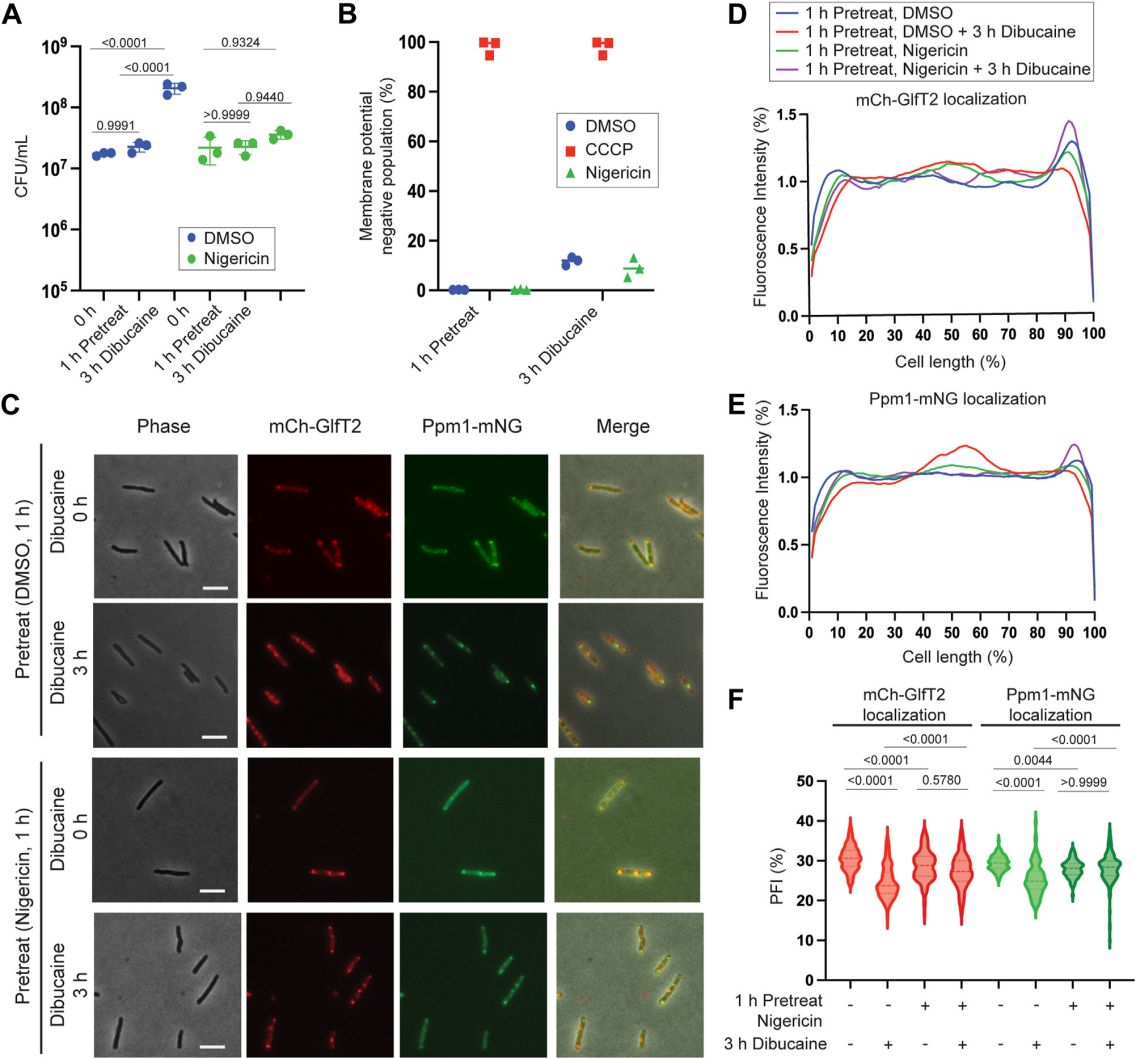
**The proton gradient rather than membrane potential is critical for the dibucaine-induced IMD de-partitioning**. *A*, CFUs, showing the survival of *M. smegmatis* after 1 h pretreatment with 5 μM nigericin followed by 3-h treatment with dibucaine. Data for the DMSO-treated cells are reproduced from Figure 4*A* as the experiment was done at the same time as Figure 4*A* experiment. *p* values were determined using two-way ANOVA followed by Tukey's multiple comparison test. *B*, flow cytometry analysis, showing that nigericin does not compromise membrane potential. See Figure 4 legend and methods for details. Data for the CCCP- and DMSO-treated cells were reproduced from Figure 4*C* as the experiment was done together with Figure 4*C* experiment. *C,* IMD markers do not de-partition upon dibucaine treatment when cells were pretreated with 5 μM nigericin. Scale bars, 5 μm. *D and E*, fluorescence intensity profiles of mCherry-GlfT2 (*D*) and Ppm1-mNeonGreen (*E*) quantified by Oufti. N = 100. Profiles of DMSO-treated cells are reproduced from Figure 4, *E* and *F*. *F*, PFI with and without nigericin pretreatment before and after dibucaine treatment. *p* values were determined by Kruskal-Wallis test followed by Dunn's multiple-comparison test.

Since we observed that disruption of the transmembrane potential does not effectively prevent dibucaine-induced movement of mCherry-GlfT2, we next treated the cells with nigericin, an ionophore that exchange K^+^(potassium) ions for H^+^ (protons) across the plasma membrane (21, 22, 23), and has been used in mycobacteria (24, 25). Because of the stoichiometric exchange of K^+^ and H^+^, nigericin disrupts ΔpH but maintains ΔΨ. Pretreatment of cells with 5 μM nigericin for 1 h, followed by 3-h dibucaine treatment, did not affect the viability of the cells (Fig. 8*A*). We used an *M. smegmatis* strain expressing a pH-sensitive ratiometric GFP and determined that 5 μM nigericin acidified the intracellular pH to 6.11 when external pH was 6.65, indicating a dissipation of the proton gradient (Table 4). However, as predicted, the transmembrane potential remained intact and comparable to the no-drug control, as assessed by the DiOC2(3) membrane potential probe (Fig. 8*B*).

**Table 4:** Nigericin collapses ΔpH

	Intracellular pH	
	DMSO	Nigericin
1 h Pre-Treatment	6.74 ± 0.05	6.11 ± 0.005
3 h Dibucaine Treatment	7.09 ± 0.01	6.20 ± 0.01

The intracellular pH before and after nigericin and dibucaine treatment was measured in a buffered medium (pH 6.65) using pH-sensitive ratiometric GFP. The internal pH of cells treated with 5 μM nigericin was reduced compared with DMSO-treated cells, indicating a collapsed proton gradient.

We next examined the localization of IMD marker proteins, mCherry-GlfT2 and Ppm1-mNeonGreen. After 1 h of nigericin treatment, IMD localization was subpolar. Even after dibucaine treatment, the sub-polar IMD localization was unaffected. In contrast, as before, without nigericin treatment (DMSO vehicle control), dibucaine induced IMD delocalization (Fig. 8, *C*–*F*). These results indicate that the proton gradient (ΔpH) rather than the transmembrane potential (ΔΨ) drives the delocalization of the IMD under dibucaine-induced membrane fluidization stress.

### Differential intracellular accumulation of dibucaine does not explain the lack of IMD redistribution

**Figure 9:**
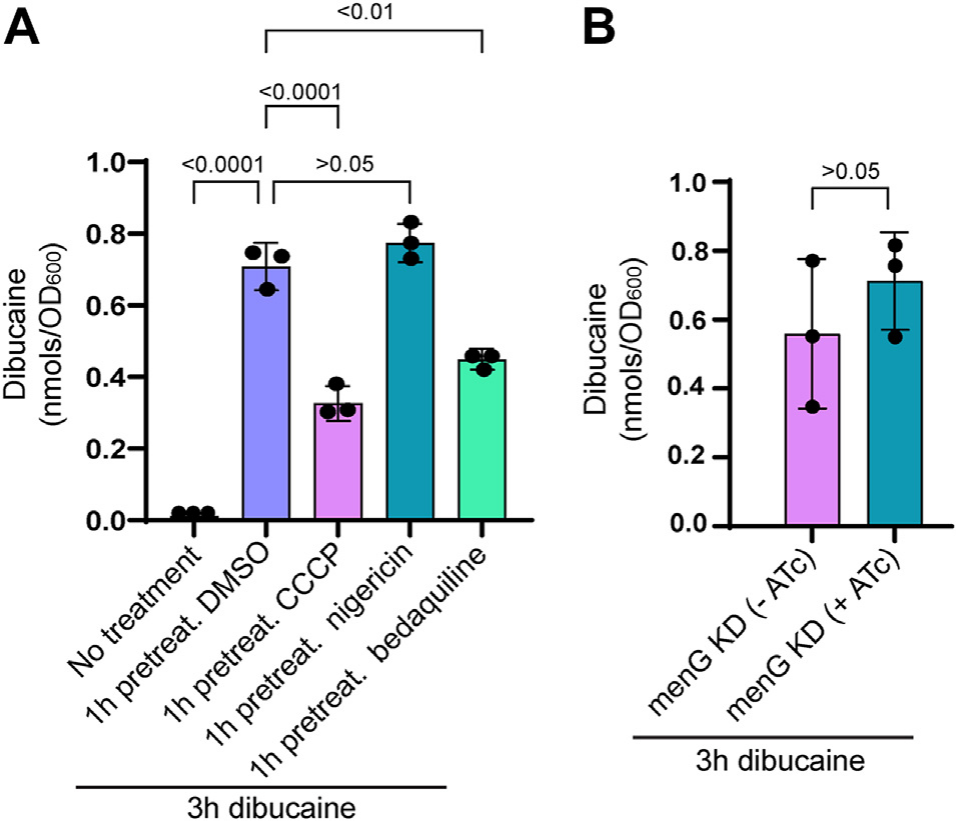
**Intracellular accumulation of dibucaine differs across drug treatments and under *menG* depletion.***A*, dibucaine accumulation in cells with no drug treatment and pre-treatment with DMSO, 10 μM CCCP, 5 μM nigericin, 14.4 nM bedaquiline followed by 3-h treatment with dibucaine. *B*, dibucaine accumulation in CRISPRi *menG* knockdown strain with and without 50 ng/ml ATc followed by 3-h dibucaine treatment. The experiment was performed in biological triplicates and *p* values were determined by the one-way ANOVA with Bonferroni *post hoc* test. values were determined by the one-way ANOVA with Bonferroni *post hoc* test.

It is possible that a proton gradient is necessary for taking up dibucaine and the pretreatment with a proton gradient disruptor results in reduced accumulation of dibucaine in the cell. To test this possibility, we pretreated cells with proton gradient disruptors and then treated them with dibucaine for 3 h. We then extracted dibucaine from harvested cells and quantified it using mass spectrometry. We found 0.33 or 0.45 nmols of dibucaine per 1 OD_600_ unit of cells accumulating in CCCP or bedaquiline-treated cells, respectively, which significantly differed from 0.71 nmols/OD_600_ accumulating in DMSO-pretreated vehicle control cells (Fig. 9*A*). However, the levels of intracellular dibucaine in nigericin-pretreated cells were comparable to that of DMSO-pretreated cells (Fig. 9*A*). Since both CCCP and nigericin blocked the dibucaine-induced redistribution of the IMD, these concentration changes in intracellular dibucaine do not explain the lack of IMD redistribution. Furthermore, we tested the *menG* knockdown strain with or without ATc, followed by dibucaine treatment. In both conditions, the levels of dibucaine accumulated at similar levels, indicating *menG* knockdown did not affect dibucaine uptake (Fig. 9*B*). Taken together, these results indicate that reduced levels of subpolar IMD de-partitioning under respiratory-deficient conditions is not due to reduced dibucaine accumulation, suggesting a more direct role of the proton gradient in IMD dynamics.

## Discussion

In this study, we showed that the spatial rearrangement of the IMD, a mycobacterial membrane domain, is dependent on the proton gradient across the inner membrane. We have previously shown that the subpolarly enriched IMD relocates to the sidewall when the cells are exposed to stress conditions such as starvation, peptidoglycan defects, and membrane fluidization (1, 5, 9, 10). Until now, our working hypothesis was that the sub-polar concentration of the IMD is an active process, and the IMD will passively diffuse and redistribute throughout the entire inner membrane of the cell once the active enrichment process is disrupted by a stressor like dibucaine. Unexpectedly, our current study argues against such a hypothesis and supports a model in which stress-induced de-partitioning of sub-polarly enriched IMD is an active process requiring a proton gradient.

The IMD delocalizes from the sub-polar region upon inhibition of cell wall biosynthesis (9), and cellular ATP levels increase when cell wall biosynthesis is inhibited by antibiotics (26). Therefore, it seemed reasonable to speculate that ATP, produced from PMF, drives the lateral movement of the IMD. Such a hypothesis could explain why the IMD was present at the sub-polar region when the proton gradient was disrupted. However, when ATP production was directly inhibited—either through CRISPRi knockdown of a H^+^-ATPase subunit or by using bedaquiline, a potent mycobacterial H^+^-ATPase inhibitor—the dibucaine-induced de-partitioning of IMD was less severely affected compared to when the proton gradient was directly disrupted by CCCP or *menG* knockdown. These observations suggest that ATP may not be the direct source of energy to mobilize the IMD. Disruption of proton gradient by CCCP affects both ΔpH and ΔΨ components of the PMF, but nigericin, which disrupts ΔpH but not ΔΨ, also blocked the dibucaine-induced de-partitioning of the IMD, suggesting that IMD de-partitioning is dependent on ΔpH. These findings offer crucial insights into bioenergetics, which drives the delocalization of the membrane domain from the sub-polar region.

We are not aware of membrane domain dynamics that are dependent on a proton gradient in any biological systems. However, spatiotemporal rearrangement of a membrane protein is dependent on a proton gradient in *Escherichia coli*. A respiratory nitrate reductase, which uses nitrogen oxides as electron acceptors under anaerobic respiration, concentrates at the poles of the rod-shaped cells when grown anaerobically (27). Presumably, the enrichment of nitrate reductase and other respiratory enzymes in a confined membrane region can enhance the efficiency of anaerobic respiration. In contrast, under aerobic growth, the enzyme disperses throughout the cell membrane. Notably, CCCP blocks the polar enrichment of nitrate reductase when oxygen is depleted, highlighting the role of the proton gradient in controlling the membrane protein localization. Analogous physiological significance may be conceivable for the mycobacterial IMD as it is a biosynthetic hub concentrating cell envelope biosynthetic enzymes at the sub-polar region of the cell, likely facilitating enzymatic reactions at the location where the cells undergo polarly restricted cell envelope growth.

How the proton gradient translates into the spatial rearrangement of the IMD remains speculative. The secondary active transporters, such as the major facilitator superfamily (MFS) and the resistance-nodulation-cell division (RND) superfamily transporters, are often proton-coupled symporters and antiporters. Na^+^, K^+^/H^+^ antiporters such as NhaA in bacteria are implicated in maintaining pH homeostasis which is important for the function of many other transporters that use Na^+^ or H^+^ as a counter ion (28, 29). In eukaryotes, local acidification by the activity of a Na^+^/H^+^ exchanger results in the protonation of a specific phosphoinositide species (phosphatidylinositol 4,5-bisphosphate), facilitating its binding to the Na^+^/H^+^ exchanger and further modulating the exchanger activity (30). Interestingly, Tn-seq analysis on genes required for survival and recovery after membrane fluidization stress identified two transporters, NhaA (MSMEG_2775) and putative monovalent cation/H^+^ antiporter subunit D (MSMEG_0846c) as significant hits (1). Therefore, it is tempting to speculate that local changes in pH modulate lipid behaviors, directly impacting the membrane domain organization. In mycobacteria, perhaps the most studied example of a proton-coupled transporter is MmpL3, which is a member of the RND superfamily and uses a proton gradient to energize the export of trehalose monomycolate (31, 32). An alternative molecular mechanism is the localized recruitment and flipping of membrane lipids by a similar type of proton-coupled lipid transporter, allowing the movement of the IMD. Finally, it remains possible that the physical movement of IMD is driven by simple diffusion, but a proton gradient is needed for “freeing” the IMD from physical confinement at the sub-polar regions.

In this study, we showed that proton gradient controls the IMD de-partitioning, but the dynamic rearrangement of subcellular IMD location may in turn ensure robust respiratory chain reactions in subcellular locations where they are needed. The IMD is the site of many biosynthetic reactions involving polyprenol-linked lipid intermediates, including the biosynthesis of menaquinones (13). Polyprenols are membrane-fluidizing and their production may be tightly regulated (2). The enzyme Cfa that converts unsaturated oleic acid to saturated, methyl-branched tuberculostearic acid is enriched in the IMD and important for the regulation of IMD dynamics (10). These observations imply that the reactions enriched in the IMD are pivotal for controlling the membrane fluidity and ensuring the spatial integrity of the plasma membrane. Interestingly, membrane fluidity correlates with respiration in evolutionarily diverse organisms. In eukaryotes, there is a direct link between respiration rates and homeoviscous adaptation of the mitochondrial inner membrane. Flux of the electron transport chain is enhanced by nutrient deprivation or treatment with a proton ionophore like carbonyl cyanide-*p*-trifluoromethoxyphenylhydrazone, and the enhanced respiration is associated with an increase in the fluidity of the inner mitochondrial membrane (33). In prokaryotes, an engineered *E. coli* strain, in which membrane fluidity is manipulated by modifying the content of unsaturated and branched fatty acids, revealed that membrane fluidity drives diffusion-limited reactions of electron transport chain enzymes (34). It is conceivable that membrane fluidity and enzymatic reaction efficiency within a membrane are tightly linked. The need of a proton gradient across the inner membrane emphasizes the importance of positioning the IMD within the cell in order to maintain the functions of the inner membrane under stress exposure.

## Experimental procedures

### Growth and chemical treatments

*M. smegmatis* mc^2^155 was grown at 37 °C in Middlebrook 7H9 broth supplemented with 11 mM glucose, 14.5 mM NaCl, 0.2% (v/v) glycerol, and 0.05% (v/v) Tween-80. CCCP (Acros), nigericin (Sigma-Aldrich), valinomycin (Tocris), and bedaquiline (Ambeed) were added as pre-treatment for 1 h after the cells reached a log phase and an equivalent volume of DMSO was used as a vehicle control. After the pre-treatment, dibucaine (Sigma-Aldrich) was added at the final concentration of 200 μg/ml. Benzyl alcohol (Sigma-Aldrich) was added at a final concentration of 100 mM for 1 h. Isoniazid (Sigma-Aldrich) was added at a final concentration of 50 μg/ml for 5.5 h d-cycloserine (Sigma-Aldrich) was added at a final concentration of 40 μg/ml for 6 h. CFUs were determined by serially diluting cell culture using Middlebrook 7H9 broth and spotting 5 μl on Middlebrook 7H10 agar supplemented with 11 mM glucose, 14.5 mM NaCl, and 0.5% (v/v) glycerol. The agar plates were incubated at 37 °C for 2 to 3 days before counting the number of microcolonies.

### Live cell imaging

Cells were grown to log phase (OD_600_ = 0.5∼1.0). After drug treatments, 5 or 10 μl of cell culture was placed on a 1% (w/v in Middlebrook 7H9) agar pad on a slide glass, and fluorescent protein localization was visualized using Nikon Eclipse Ti2 Widefield Microbe System (objective lens, 100x oil immersion, N.A., 1.45). All fluorescence images were taken at the exposure of 200 ms for mCherry fluorescence and 160 ms for mNeonGreen fluorescence with a gain of two. Fluorescence intensity profiles were quantified as before (1). Briefly, cell shape was contoured using Oufti (35), and each cell was divided into 100 sections along the long axis. Average relative fluorescence intensity was calculated using MATLAB with published scripts (36) and plotted along the normalized cell length. The polarity distribution was calculated by the percentage of signal associated with the distal 15% of rod-shaped cells as quantified previously (1). Statistical analysis was conducted using GraphPad Prism 9 (version 9.4.0). *p* values were calculated using Kruskal-Wallis test, followed by Dunn's multiple comparison test, and *p* < 0.05 was considered statistically significant.

### Preparation of recombinant strains

*M. smegmatis* expressing both mCherry-GlfT2 and Ppm1-mNeonGreen from their endogenous loci was previously established (3). *M. smegmatis* CRISPRi depletion strains were created using plasmids obtained from MSRdb (12, 37). The CRISPRi vectors for each gene *menG*, *atpD* and *qcrAB* were introduced *via* mycobacteriophage L5 *attB* integration into the IMD marker strain expression mCherry-GlfT2 and Ppm1-mNeonGreen.

### Flow cytometry

Five mL of log phase *M. smegmatis* cells were treated with respiratory chain inhibitors and further treated with 200 μg/ml dibucaine. For valinomycin treatment, the cells were first adjusted to an OD of 0.3 in Middlebrook 7H9 and treated with 22 μM valinomycin in the presence of 200 mM KCl. DiOC_2_(3) (TCI) was added to a 2-ml aliquot of the cell to the final concentration of 30 μM. After a 15-min incubation, cells were centrifuged at 2000*g* for 5 min and the pellet was resuspended in 2% formaldehyde in PBS for fixation. For valinomycin treatment, cells were fixed in 2% formaldehyde in PBS supplemented with 200 mM KCl. Fixed cells were washed again and resuspended in PBS for flow cytometry analyses using a three-laser (405 nm, 488 nm and 640 nm) Dual LSRFortessa (BD). As in a previous report (38), DiOC_2_(3) was excited at 488 nm with red emission detected through one filter set (a long pass (LP) filter 600 nm and band pass (BP) filter 610 nm/20 nm), and green emission detected through another filter set (LP filter 505 nm and BP filter 525 nm/50 nm). As a positive control for the disruption of membrane potential, cells were incubated with 25 μM CCCP (Sigma-Aldrich) for 1 h. Data were analyzed using FlowJo 10.0 (BD). The red/green ratio of DiOC_2_(3) was determined using the Derived Parameter function comparing the DiOC_2_(3) red and green median fluorescence parameters.

### Cellular ATP measurements

The ATP content of bacterial cultures was quantified using the CellTiter-Glo 2.0 Cell Viability Assay kit (Promega, G9241). Log phase cells were pre-treated with CCCP, nigericin, or bedaquiline for 1 h, followed by a 3-h treatment with dibucaine. After incubation, the samples were mixed with a two-fold volume of Tris-EDTA buffer (100 mM Tris-HCl, 4 mM EDTA, pH 7.75) and heated at 100 °C for 5 min. The samples were then centrifuged, and the supernatants were collected. Each supernatant (50 μl) was mixed with an equal volume of CellTiter-Glo reagent, incubated for 2 min with shaking, and kept in a white 96-well plate (ThermoFisher) at room temperature in the dark for 10 min to stabilize luminescence signal. Luminescence was subsequently recorded using a Synergy 2 Multi-Mode Plate Reader (BioTek).

### Methylene blue respiration assay

One OD_600_ unit of each bacterial culture was resuspended in 0.001% methylene blue solution within a plastic cuvette. To create an oxygen barrier, 500 μl of mineral or vegetable oil was layered on top of the culture. The cuvette was further sealed with a lid and parafilm. After incubating for 30 min, the absorbance was measured at 665 nm using a Nanodrop Microvolume Spectrophotometer (ThermoFisher).

### Intracellular pH measurement

The pH-GFP plasmid, pUV15-pHGFP, was obtained from Addgene and electroporated into wild-type *M. smegmatis* cells. The plasmid integrates into the genome *via* L5 *attB* site as described (39). For standard curve generation, protein concentration of a cell lysate from the strain containing the pH-GFP construct was determined by the bicinchoninic acid assay (ThermoFisher) and a volume of lysate containing 100 μg of protein was suspended in 100 μl of phosphate-citrate buffer, with pH values ranging from 5.5 to 8.5. For measuring intracellular pH, cells harboring the pH-GFP plasmid were grown to the log phase, subjected to appropriate drug treatments, and concentrated 30 times to enhance the fluorescence signal. Fluorescence was measured by exciting the cells at two wavelengths, 360/40 and 485/20 nm, with emission detected at 510/20 nm using a Synergy 2 Multi-Mode Plate Reader (BioTek). The ratio of fluorescence from 485/510 and 360/510 was plotted against the standard curve to determine intracellular pH.

### RNA purification

RNA was extracted and purified from the cell lysate as previously described (40). Cell culture was centrifuged at 3220*g* for 10 min. The wet pellets (∼400 mg) were then frozen at −80 °C overnight and then resuspend in 1 ml of TRIzol (Fisher Scientific). Cells were lysed with 0.5 ml of DNAse/RNAse free zirconia beads using a BeadBug microtube homogenizer (Benchmark Scientific) at 4 °C with beating at 4000 rpm for 1 min, followed by resting on ice for 2 min. The bead homogenization was performed three times in total and followed by a 5-min incubation at room temperature. The samples were then centrifuged for 30 s at 10,000*g* at 4 °C. The purified RNA was then concentrated using an RNA Clean and Concentrator kit by following the manufacturer's protocol (Zymo Research). Two micrograms of RNA were used to synthesize cDNA using Maxima H minus reverse transcriptase (ThermoFisher) and 1 μM random primers (ThermoFisher) in accordance with the manufacturer's instructions.

### Quantitative real-time PCR

For each 25-μl reaction mixture, 12.5 μl of the FastStart Universal SYBR Green Master mix (Roche) was mixed with cDNA and primers to achieve the final concentrations of 100 ng/μl and 400 nM, respectively. For a housekeeping gene, we used *gyrB* (MSMEG_0005) as before (5′-TATTCGGAGTCGGTGCACAC-3′ and 5′- CTTGTCCTTGGCATACCGGT-3′) (41). To amplify *menG* (MSMEG_1115), *qcrC* (MSMEG_4261), and *atpD* (MSMEG_4936), we used the following primer pairs, respectively: 5′-AACGTCGTCGACCACAAG-3′ and 5′-CTTGTAGACCGTGGAGAACAG-3′; 5′-CAAGTACGCACCTGACCTC-3′ and 5′-ATGTCGCGCTTCTCATCC-3′; and 5′-TTGGTGTTCGGTCAGATGG-3′ and 5′-CCGGAAGATGTTGTCGATGA-3′. Quantitative PCR was performed on the Bio-RAD CFX96 Touch Real-Time PCR Detection System with the following program: 95 °C for 10 min, followed by 40 cycles of incubations at 95 °C for 30 s, 62 °C for 30 s, and 72 °C for 30 s. Data were analyzed using Bio-Rad CFX Manager 3.1 to determine the 2^–ΔΔ*CT*^ (where *CT* is the threshold cycle).

Quantification of intra-bacterial dibucaine accumulation by liquid chromatography-mass spectrometry (LC-MS)

Log phase cells were treated with different drug conditions and end time points were collected in triplicates. One mL of each collected sample was centrifuged at 14,500*g* for 10 min and resuspended in 1 ml 0.85% NaCl (w/v in water). The cells were pelleted at 14,500*g* for 10 min and the supernatant was discarded. The pellet was resuspended in 1 ml of methanol: acetonitrile: water (2:2:1, v/v/v). The cells were lysed by bead beating using lysis matrix B (MP) in BeadBug microtube homogenizer (Benchmark Scientific) at 4 °C with beating at 4000 rpm for six cycles with 2 min on ice between cycles. The vials were centrifuged at 14,500*g* for 10 to 15 min at 4 °C and 0.7 ml of the supernatant was passed through a 0.22 μm Spin-X centrifuge filter (Costar). The filtrate was analyzed by LC-MS as described below.

A Poroshell 120 EC-C18 column (2.1 x 50 mm; 1.9 μ particle size) and an Agilent 1290 Infinity II HPLC system coupled to an Agilent 6125 single quadrupole mass spectrometer, featuring an API-ES ion source with positive polarity, were used to analyze the samples. The solvent system of water/acetonitrile acidified with 0.1% formic acid was used with a conventional gradient (represented as the percentage of acetonitrile): 0 to 0.4 min, 5%; 0.4 to 0.5 min, 5% to 30%; 0.5 to 3.0 min, 30% to 95%; 3.0 to 3.3 min, 95%; 3.3 to 3.6 min, 95% to 5%. All samples were assayed with a flow rate of 0.5 ml/min and a 1 min post-run sequence to equilibrate the system in between runs. A scan mode ranging from m/z 100 to 1000 was first used to determine the retention times of dibucaine and the internal standard (verapamil). Subsequently, the selective ion monitoring (SIM) mode was used to increase the sensitivity for the quantification of dibucaine and verapamil. The calibration curve for standard mixtures of the dibucaine in MAW was constructed by using dibucaine concentrations ranging from 5 to 0.002 μM in the presence of 50 nM verapamil. The lower limit of detection (LLOD) was determined for dibucaine as the lowest concentration of the analyte that could be distinguished from the baseline so that the analyte peak height was at least twice the height of the baseline. Dibucaine/verapamil peak area ratio was plotted *versus* the dibucaine concentration to enable the determination of the calibration factor (slope) of the fitted line using the linear-least squares approach. Different dibucaine calibration curves in the presence of 10 μM CCCP, 5 μM nigericin, or 14.4 nM bedaquiline were also obtained to demonstrate a lack of effect of these compounds on dibucaine ionization. For sample analysis, 0.25 ml of flow-through post-filtration was supplemented with a constant concentration of verapamil (50 nM) followed by vortexing, and 10 μl of the sample was injected into the LC-MS. The extracted peak areas of dibucaine and verapamil for each sample were quantified. The dibucaine/verapamil peak area ratio was then divided by the calibration factor and then further multiplied by 1000 to get values in nM, and then divided by sample volume (0.001 ml) to obtain concentrations in nmol. The OD_600_ for each sample was used to normalize the intrabacterial accumulation to provide units of nmols/OD_600_ (42).

## Data availability

All data underlying this article are either contained within the manuscript or will be shared on reasonable request to the corresponding author.

## Conflict of interest

The authors declare that they have no conflicts of interest with the contents of this article.

## References

[1] Kado T., Akbary Z., Motooka D., Sparks I.L., Melzer E.S., Nakamura S. (2023). A cell wall synthase accelerates plasma membrane partitioning in mycobacteria. eLife.

[2] Hayashi J.M., Morita Y.S. (2019). Mycobacterial membrane domain, or a primordial organelle?. Yale J. Biol. Med..

[3] Hayashi J.M., Luo C.-Y., Mayfield J.A., Hsu T., Fukuda T., Walfield A.L. (2016). Spatially distinct and metabolically active membrane domain in mycobacteria. Proc. Natl. Acad. Sci. U. S. A.

[4] Morita Y.S., Velasquez R., Taig E., Waller R.F., Patterson J.H., Tull D. (2005). Compartmentalization of lipid biosynthesis in mycobacteria. J. Biol. Chem..

[5] García-Heredia A., Kado T., Sein C.E., Puffal J., Osman S.H., Judd J. (2021). Membrane-partitioned cell wall synthesis in mycobacteria. eLife.

[6] Rokicki C.A.Z., Brenner J.R., Dills A.H., Judd J.J., Kester J.C., Puffal J. (2021). Fluorescence imaging-based discovery of membrane domain-associated proteins in *Mycobacterium smegmatis*. J. Bacteriol..

[7] Savková K., Danchenko M., Fabianová V., Bellová J., Bencúrová M., Huszár S. (2024). Compartmentalization of galactan biosynthesis in mycobacteria. J. Biol. Chem..

[8] Zhu J., Wolf I.D., Dulberger C.L., Won H.I., Kester J.C., Judd J.A. (2021). Spatiotemporal localization of proteins in mycobacteria. Cell Rep..

[9] Hayashi J.M., Richardson K., Melzer E.S., Sandler S.J., Aldridge B.B., Siegrist M.S. (2018). Stress-induced reorganization of the mycobacterial membrane domain. mBio.

[10] Prithviraj M., Kado T., Mayfield J.A., Young D.C., Huang A.D., Motooka D. (2023). Tuberculostearic acid controls mycobacterial membrane compartmentalization. mBio.

[11] Sinensky M. (1974). Homeoviscous adaptation--a homeostatic process that regulates the viscosity of membrane lipids in *Escherichia coli*. Proc. Natl. Acad. Sci. U. S. A.

[12] Bosch B., DeJesus M.A., Poulton N.C., Zhang W., Engelhart C.A., Zaveri A. (2021). Genome-wide gene expression tuning reveals diverse vulnerabilities of M. tuberculosis. Cell.

[13] Puffal J., Mayfield J.A., Moody D.B., Morita Y.S. (2018). Demethylmenaquinone methyl transferase is a membrane domain-associated protein essential for menaquinone homeostasis in *Mycobacterium smegmatis*. Front. Microbiol..

[14] Dragset M.S., Ioerger T.R., Zhang Y.J., Mærk M., Ginbot Z., Sacchettini J.C. (2019). Genome-wide phenotypic profiling identifies and categorizes genes required for mycobacterial low iron fitness. Sci. Rep..

[15] Sone N., Nagata K., Kojima H., Tajima J., Kodera Y., Kanamaru T. (2001). A novel hydrophobic diheme c-type cytochrome. Purification from *Corynebacterium glutamicum* and analysis of the QcrCBA operon encoding three subunit proteins of a putative cytochrome reductase complex. Biochim. Biophys. Acta.

[16] Matsoso L.G., Kana B.D., Crellin P.K., Lea-Smith D.J., Pelosi A., Powell D. (2005). Function of the cytochrome bc1-aa3 branch of the respiratory network in mycobacteria and network adaptation occurring in response to its disruption. J. Bacteriol..

[17] Andries K., Verhasselt P., Guillemont J., Göhlmann H.W.H., Neefs J.-M., Winkler H. (2005). A diarylquinoline drug active on the ATP synthase of *Mycobacterium tuberculosis*. Science.

[18] Guo H., Courbon G.M., Bueler S.A., Mai J., Liu J., Rubinstein J.L. (2021). Structure of mycobacterial ATP synthase bound to the tuberculosis drug bedaquiline. Nature.

[19] Brown-Elliott B.A., Wallace R.J. (2019). In vitro susceptibility testing of bedaquiline against *Mycobacterium abscessus* complex. Antimicrob. Agents Chemother..

[20] Chen C., Gardete S., Jansen R.S., Shetty A., Dick T., Rhee K.Y. (2018). Verapamil targets membrane energetics in *Mycobacterium tuberculosis*. Antimicrob. Agents Chemother..

[21] Harned R.L., Hidy P.H., Corum C.J., Jones K.L. (1951). Nigericin a new crystalline antibiotic from an unidentified *Streptomyces*. Antibiot. Chemother. (Northfield).

[22] Pressman B.C. (1976). Biological applications of ionophores. Annu. Rev. Biochem..

[23] Reed P.W. (1979). Ionophores. Methods Enzymol..

[24] Beites T., O'Brien K., Tiwari D., Engelhart C.A., Walters S., Andrews J. (2019). Plasticity of the *Mycobacterium tuberculosis* respiratory chain and its impact on tuberculosis drug development. Nat. Commun..

[25] Jeon A.B., Ackart D.F., Li W., Jackson M., Melander R.J., Melander C. (2019). 2-aminoimidazoles collapse mycobacterial proton motive force and block the electron transport chain. Sci. Rep..

[26] Shetty A., Dick T. (2018). Mycobacterial cell wall synthesis inhibitors cause lethal ATP burst. Front. Microbiol..

[27] Alberge F., Espinosa L., Seduk F., Sylvi L., Toci R., Walburger A. (2015). Dynamic subcellular localization of a respiratory complex controls bacterial respiration. eLife.

[28] Padan E., Tzubery T., Herz K., Kozachkov L., Rimon A., Galili L. (2004). NhaA of *Escherichia coli*, as a model of a pH-regulated Na^+^/H^+^ antiporter. Biochim. Biophys. Acta.

[29] Vinothkumar K.R., Smits S.H.J., Kühlbrandt W. (2005). pH-induced structural change in a sodium/proton antiporter from *Methanococcus jannaschii*. EMBO J..

[30] Abu Jawdeh B.G., Khan S., Deschênes I., Hoshi M., Goel M., Lock J.T. (2011). Phosphoinositide binding differentially regulates NHE1 Na^+^/H^+^ exchanger-dependent proximal tubule cell survival. J. Biol. Chem..

[31] Stevens C.M., Babii S.O., Pandya A.N., Li W., Li Y., Mehla J. (2022). Proton transfer activity of the reconstituted *Mycobacterium tuberculosis* MmpL3 is modulated by substrate mimics and inhibitors. Proc. Natl. Acad. Sci. U. S. A.

[32] Xu Z., Meshcheryakov V.A., Poce G., Chng S.-S. (2017). MmpL3 is the flippase for mycolic acids in mycobacteria. Proc. Natl. Acad. Sci. U. S. A.

[33] Singh G., George G., Raja S.O., Kandaswamy P., Kumar M., Thutupalli S. (2023). A molecular rotor FLIM probe reveals dynamic coupling between mitochondrial inner membrane fluidity and cellular respiration. Proc. Natl. Acad. Sci. U. S. A.

[34] Budin I., de Rond T., Chen Y., Chan L.J.G., Petzold C.J., Keasling J.D. (2018). Viscous control of cellular respiration by membrane lipid composition. Science.

[35] Paintdakhi A., Parry B., Campos M., Irnov I., Elf J., Surovtsev I. (2016). Oufti: an integrated software package for high-accuracy, high-throughput quantitative microscopy analysis. Mol. Microbiol..

[36] García-Heredia A., Pohane A.A., Melzer E.S., Carr C.R., Fiolek T.J., Rundell S.R. (2018). Peptidoglycan precursor synthesis along the sidewall of pole-growing mycobacteria. eLife.

[37] Judd J.A., Canestrari J., Clark R., Joseph A., Lapierre P., Lasek-Nesselquist E. (2021). A mycobacterial systems Resource for the research community. mBio.

[38] Novo D.J., Perlmutter N.G., Hunt R.H., Shapiro H.M. (2000). Multiparameter flow cytometric analysis of antibiotic effects on membrane potential, membrane permeability, and bacterial counts of *Staphylococcus aureus* and *Micrococcus luteus*. Antimicrob. Agents Chemother..

[39] Vandal O.H., Pierini L.M., Schnappinger D., Nathan C.F., Ehrt S. (2008). A membrane protein preserves intrabacterial pH in intraphagosomal *Mycobacterium tuberculosis*. Nat. Med..

[40] Benjak A., Sala C., Hartkoorn R.C. (2015). Whole-transcriptome sequencing for high-resolution transcriptomic analysis in *Mycobacterium tuberculosis*. Methods Mol. Biol..

[41] Rahlwes K.C., Osman S.H., Morita Y.S. (2020). Role of LmeA, a mycobacterial periplasmic protein, in maintaining the mannosyltransferase MptA and its product lipomannan under stress. mSphere.

[42] Ahn Y.-M., Lavin R.C., Tan S., Freundlich J.S. (2023). Liquid chromatography-mass spectrometry-based protocol to measure drug accumulation in *Mycobacterium tuberculosis* and its host cell. STAR Protoc..

[43] Nguyen P.P., Kado T., Prithviraj M., Siegrist M.S., Morita Y.S. (2022). Inositol acylation of phosphatidylinositol mannosides: a rapid mass response to membrane fluidization in mycobacteria. J. Lipid Res..

